# 6-Bromo-4-[2-(4-fluoro­benzyl­idene)hydrazin-1-yl­idene]-1-methyl-3,4-dihydro-1*H*-2λ^6^,1-benzothia­zine-2,2-dione

**DOI:** 10.1107/S1600536812037403

**Published:** 2012-09-05

**Authors:** Muhammad Shafiq, Islam Ullah Khan, William T. A. Harrison, Ajaz Hussain, Hina Ashraf

**Affiliations:** a Department of Chemistry, Government College University, Faisalabad 38040, Pakistan; b Materials Chemistry Laboratory, Department of Chemistry, Government College University, Lahore 54000, Pakistan; c Department of Chemistry, University of Aberdeen, Meston Walk, Aberdeen AB24 3UE, Scotland

## Abstract

In the title compound, C_16_H_13_BrFN_3_O_2_S, the dihedral angle between the aromatic rings is 2.55 (19)° and the C=N—N=C torsion angle is 178.9 (3)°. The conformation of the thia­zine ring is an envelope, with the S atom displaced by −0.811 (3) Å from the mean plane of the other five atoms (r.m.s. deviation = 0.042 Å). In the crystal, C—H⋯O inter­actions link the mol­ecules and weak aromatic π–π stacking between the fluoro­benzene and bromo­benzene rings [centroid–centroid separation = 3.720 (2) Å and inter­planar angle = 2.6 (2)°] is also observed.

## Related literature

For the synthesis and for the biological activity of related materials, see: Shafiq, Zia-Ur-Rehman *et al.* (2011 ▶). For a related structure, see: Shafiq, Khan *et al.* (2011 ▶)
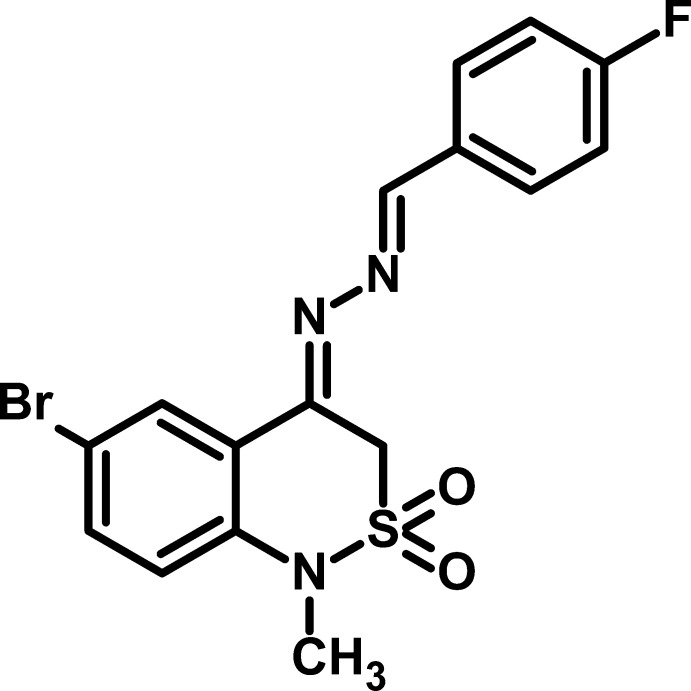


## Experimental

### 

#### Crystal data

C_16_H_13_BrFN_3_O_2_S
*M* *_r_* = 410.26
Triclinic, 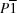

*a* = 7.8996 (4) Å
*b* = 9.0070 (4) Å
*c* = 13.5057 (7) Å
α = 104.176 (3)°
β = 90.977 (3)°
γ = 113.466 (3)°
*V* = 847.51 (7) Å^3^
*Z* = 2
Mo *K*α radiation
μ = 2.57 mm^−1^
*T* = 296 K
0.37 × 0.16 × 0.14 mm

#### Data collection

Bruker APEXII CCD diffractometer
Absorption correction: multi-scan (*SADABS*; Bruker, 2007 ▶) *T*_min_ = 0.450, *T*_max_ = 0.715
17622 measured reflections
4186 independent reflections
2331 reflections with *I* > 2σ(*I*)
*R*_int_ = 0.038

#### Refinement

*R*[*F* ^2^ > 2σ(*F* ^2^)] = 0.043
*wR*(*F* ^2^) = 0.118
*S* = 0.99
4186 reflections
218 parameters
H-atom parameters constrained
Δρ_max_ = 0.58 e Å^−3^
Δρ_min_ = −0.78 e Å^−3^

### 

Data collection: *APEX2* (Bruker, 2007 ▶); cell refinement: *SAINT* (Bruker, 2007 ▶); data reduction: *SAINT*; program(s) used to solve structure: *SHELXS97* (Sheldrick, 2008 ▶); program(s) used to refine structure: *SHELXL97* (Sheldrick, 2008 ▶); molecular graphics: *ORTEP-3* (Farrugia, 1997 ▶); software used to prepare material for publication: *SHELXL97*.

## Supplementary Material

Crystal structure: contains datablock(s) global, I. DOI: 10.1107/S1600536812037403/kj2209sup1.cif

Structure factors: contains datablock(s) I. DOI: 10.1107/S1600536812037403/kj2209Isup2.hkl

Supplementary material file. DOI: 10.1107/S1600536812037403/kj2209Isup3.cml

Additional supplementary materials:  crystallographic information; 3D view; checkCIF report

## Figures and Tables

**Table 1:** Hydrogen-bond geometry (Å, °)

*D*—H⋯*A*	*D*—H	H⋯*A*	*D*⋯*A*	*D*—H⋯*A*
C8—H8*B*⋯O2^i^	0.97	2.56	3.397 (4)	145
C9—H9⋯O1^ii^	0.93	2.39	3.292 (4)	163

Symmetry codes: (i) 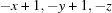; (ii) 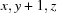.

**Fig. 1.**
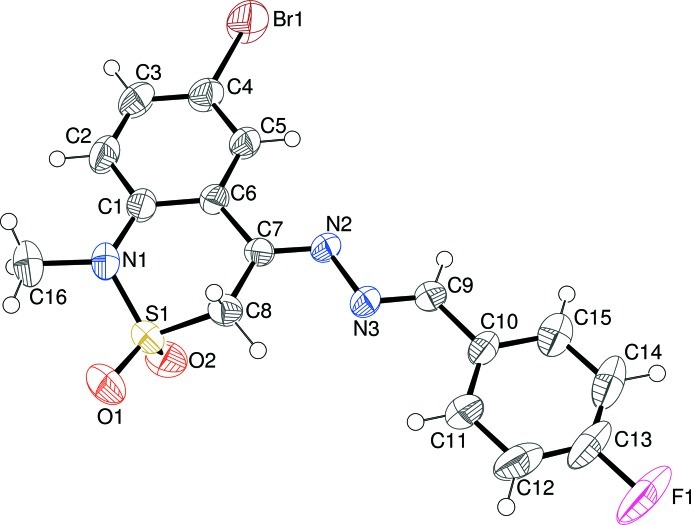
The molecular structure of the title compound, showing displacement ellipsoids at the 50% probability level.

## supplementary crystallographic information

### Experimental

For the synthesis, see: Shafiq, Zia-Ur-Rehman *et al.* (2011). Yellow needles were recrystallized from ethylacetate under slow evaporation.

### Refinement

The H atoms were placed in calculated positions (C—H = 0.93–0.97 Å) and refined as riding. The methyl group was allowed to rotate, but not to tip, to best fit the electron density. The constraint *U*_iso_(H) = 1.2*U*_eq_(C) or 1.5*U*_eq_(methyl C) was applied.

### Crystal data

**Table:**

C_16_H_13_BrFN_3_O_2_S	*Z* = 2
*M**_r_* = 410.26	*F*(000) = 412
Triclinic, *P*1	*D*_x_ = 1.608 Mg m^−^^3^
Hall symbol: -P 1	Mo *K*α radiation, λ = 0.71073 Å
*a* = 7.8996 (4) Å	Cell parameters from 4416 reflections
*b* = 9.0070 (4) Å	θ = 2.6–22.8°
*c* = 13.5057 (7) Å	µ = 2.57 mm^−^^1^
α = 104.176 (3)°	*T* = 296 K
β = 90.977 (3)°	Needle, yellow
γ = 113.466 (3)°	0.37 × 0.16 × 0.14 mm
*V* = 847.51 (7) Å^3^	

### Data collection

**Table:**

Bruker APEXII CCD diffractometer	4186 independent reflections
Radiation source: fine-focus sealed tube	2331 reflections with *I* > 2σ(*I*)
Graphite monochromator	*R*_int_ = 0.038
ω scans	θ_max_ = 28.3°, θ_min_ = 2.6°
Absorption correction: multi-scan (*SADABS*; Bruker, 2007)	*h* = −10→10
*T*_min_ = 0.450, *T*_max_ = 0.715	*k* = −12→12
17622 measured reflections	*l* = −17→17

### Refinement

**Table:**

Refinement on *F*^2^	Primary atom site location: structure-invariant direct methods
Least-squares matrix: full	Secondary atom site location: difference Fourier map
*R*[*F*^2^ > 2σ(*F*^2^)] = 0.043	Hydrogen site location: inferred from neighbouring sites
*wR*(*F*^2^) = 0.118	H-atom parameters constrained
*S* = 0.99	*w* = 1/[σ^2^(*F*_o_^2^) + (0.0479*P*)^2^ + 0.5245*P*] where *P* = (*F*_o_^2^ + 2*F*_c_^2^)/3
4186 reflections	(Δ/σ)_max_ = 0.001
218 parameters	Δρ_max_ = 0.58 e Å^−^^3^
0 restraints	Δρ_min_ = −0.78 e Å^−^^3^

### Special details

**Table:**

Geometry. All e.s.d.'s (except the e.s.d. in the dihedral angle between two l.s. planes) are estimated using the full covariance matrix. The cell e.s.d.'s are taken into account individually in the estimation of e.s.d.'s in distances, angles and torsion angles; correlations between e.s.d.'s in cell parameters are only used when they are defined by crystal symmetry. An approximate (isotropic) treatment of cell e.s.d.'s is used for estimating e.s.d.'s involving l.s. planes.
Refinement. Refinement of *F*^2^ against ALL reflections. The weighted *R*-factor *wR* and goodness of fit *S* are based on *F*^2^, conventional *R*-factors *R* are based on *F*, with *F* set to zero for negative *F*^2^. The threshold expression of *F*^2^ > σ(*F*^2^) is used only for calculating *R*-factors(gt) *etc*. and is not relevant to the choice of reflections for refinement. *R*-factors based on *F*^2^ are statistically about twice as large as those based on *F*, and *R*- factors based on ALL data will be even larger.

### Fractional atomic coordinates and isotropic or equivalent isotropic displacement parameters (Å^2^)

**Table:**

	*x*	*y*	*z*	*U*_iso_*/*U*_eq_	
C1	0.9739 (4)	0.8291 (4)	0.2392 (2)	0.0408 (7)	
C2	1.0591 (5)	0.8914 (4)	0.3410 (2)	0.0515 (8)	
H2	1.0804	0.8194	0.3734	0.062*	
C3	1.1121 (5)	1.0568 (4)	0.3941 (2)	0.0535 (9)	
H3	1.1720	1.0973	0.4612	0.064*	
C4	1.0757 (5)	1.1622 (4)	0.3472 (2)	0.0512 (8)	
C5	0.9894 (4)	1.1042 (4)	0.2476 (2)	0.0432 (7)	
H5	0.9654	1.1771	0.2172	0.052*	
C6	0.9377 (4)	0.9379 (3)	0.1919 (2)	0.0354 (6)	
C7	0.8470 (4)	0.8820 (3)	0.0850 (2)	0.0345 (6)	
C8	0.8201 (5)	0.7111 (3)	0.0194 (2)	0.0458 (8)	
H8A	0.9351	0.7173	−0.0075	0.055*	
H8B	0.7248	0.6743	−0.0384	0.055*	
C9	0.6595 (4)	1.0158 (4)	−0.0800 (2)	0.0379 (7)	
H9	0.6818	1.1194	−0.0344	0.045*	
C10	0.5657 (4)	0.9726 (4)	−0.1840 (2)	0.0409 (7)	
C11	0.5109 (5)	0.8138 (4)	−0.2507 (2)	0.0552 (9)	
H11	0.5309	0.7307	−0.2296	0.066*	
C12	0.4260 (5)	0.7788 (6)	−0.3493 (3)	0.0735 (12)	
H12	0.3877	0.6722	−0.3948	0.088*	
C13	0.3996 (5)	0.9026 (7)	−0.3781 (3)	0.0741 (13)	
C14	0.4504 (5)	1.0597 (6)	−0.3152 (3)	0.0725 (12)	
H14	0.4300	1.1416	−0.3377	0.087*	
C15	0.5335 (5)	1.0944 (5)	−0.2165 (3)	0.0564 (9)	
H15	0.5682	1.2009	−0.1713	0.068*	
C16	0.9850 (6)	0.5539 (5)	0.2319 (3)	0.0817 (13)	
H16A	1.1167	0.6067	0.2535	0.122*	
H16B	0.9531	0.4469	0.1827	0.122*	
H16C	0.9202	0.5378	0.2906	0.122*	
S1	0.75433 (14)	0.56638 (10)	0.09239 (7)	0.0548 (3)	
N1	0.9317 (4)	0.6609 (3)	0.1849 (2)	0.0542 (7)	
N2	0.7961 (3)	0.9817 (3)	0.05209 (17)	0.0383 (6)	
N3	0.7109 (3)	0.9153 (3)	−0.05055 (17)	0.0407 (6)	
O1	0.7609 (4)	0.4128 (3)	0.0372 (2)	0.0824 (9)	
O2	0.5862 (3)	0.5604 (3)	0.1320 (2)	0.0672 (7)	
F1	0.3170 (4)	0.8672 (4)	−0.47568 (18)	0.1185 (11)	
Br1	1.14956 (8)	1.39006 (5)	0.42019 (3)	0.0926 (2)	

### Atomic displacement parameters (Å^2^)

**Table:**

	*U*^11^	*U*^22^	*U*^33^	*U*^12^	*U*^13^	*U*^23^
C1	0.0408 (18)	0.0441 (16)	0.0416 (17)	0.0197 (15)	0.0030 (14)	0.0151 (13)
C2	0.054 (2)	0.064 (2)	0.0436 (18)	0.0272 (18)	−0.0006 (15)	0.0233 (16)
C3	0.057 (2)	0.069 (2)	0.0319 (17)	0.0243 (18)	−0.0045 (15)	0.0115 (15)
C4	0.060 (2)	0.0507 (18)	0.0361 (17)	0.0231 (17)	−0.0059 (15)	0.0009 (14)
C5	0.0503 (19)	0.0433 (16)	0.0337 (15)	0.0197 (15)	−0.0039 (14)	0.0066 (13)
C6	0.0375 (17)	0.0377 (14)	0.0306 (14)	0.0149 (13)	0.0023 (12)	0.0099 (12)
C7	0.0352 (16)	0.0314 (13)	0.0329 (15)	0.0100 (13)	0.0015 (12)	0.0086 (11)
C8	0.056 (2)	0.0360 (15)	0.0395 (17)	0.0171 (15)	−0.0020 (15)	0.0041 (13)
C9	0.0393 (17)	0.0373 (15)	0.0335 (15)	0.0142 (13)	0.0010 (13)	0.0068 (12)
C10	0.0352 (17)	0.0536 (18)	0.0359 (16)	0.0182 (15)	0.0000 (13)	0.0164 (13)
C11	0.056 (2)	0.057 (2)	0.0431 (19)	0.0166 (18)	−0.0090 (16)	0.0091 (15)
C12	0.064 (3)	0.089 (3)	0.041 (2)	0.015 (2)	−0.0131 (18)	0.004 (2)
C13	0.050 (2)	0.129 (4)	0.043 (2)	0.031 (3)	−0.0069 (17)	0.034 (2)
C14	0.065 (3)	0.116 (4)	0.065 (3)	0.050 (3)	0.009 (2)	0.052 (3)
C15	0.054 (2)	0.075 (2)	0.052 (2)	0.0337 (19)	0.0086 (17)	0.0260 (18)
C16	0.102 (3)	0.059 (2)	0.096 (3)	0.042 (2)	−0.015 (3)	0.030 (2)
S1	0.0655 (6)	0.0316 (4)	0.0608 (5)	0.0150 (4)	−0.0081 (4)	0.0115 (4)
N1	0.0662 (19)	0.0447 (15)	0.0571 (17)	0.0273 (14)	−0.0093 (14)	0.0170 (13)
N2	0.0437 (15)	0.0375 (12)	0.0291 (12)	0.0151 (12)	−0.0052 (10)	0.0048 (10)
N3	0.0495 (15)	0.0401 (13)	0.0294 (12)	0.0173 (12)	−0.0045 (11)	0.0070 (10)
O1	0.113 (2)	0.0376 (13)	0.090 (2)	0.0338 (14)	−0.0166 (17)	0.0037 (12)
O2	0.0524 (16)	0.0561 (15)	0.0801 (18)	0.0050 (12)	0.0048 (13)	0.0266 (13)
F1	0.0919 (18)	0.199 (3)	0.0528 (14)	0.043 (2)	−0.0233 (13)	0.0460 (18)
Br1	0.1381 (5)	0.0639 (3)	0.0568 (3)	0.0455 (3)	−0.0405 (3)	−0.01902 (18)

### Geometric parameters (Å, º)

**Table:**

C1—C2	1.395 (4)	C10—C11	1.381 (4)
C1—C6	1.405 (4)	C10—C15	1.385 (4)
C1—N1	1.411 (4)	C11—C12	1.384 (5)
C2—C3	1.370 (5)	C11—H11	0.9300
C2—H2	0.9300	C12—C13	1.355 (6)
C3—C4	1.375 (5)	C12—H12	0.9300
C3—H3	0.9300	C13—C14	1.354 (6)
C4—C5	1.373 (4)	C13—F1	1.365 (4)
C4—Br1	1.887 (3)	C14—C15	1.382 (5)
C5—C6	1.388 (4)	C14—H14	0.9300
C5—H5	0.9300	C15—H15	0.9300
C6—C7	1.475 (4)	C16—N1	1.457 (4)
C7—N2	1.281 (3)	C16—H16A	0.9600
C7—C8	1.505 (4)	C16—H16B	0.9600
C8—S1	1.746 (3)	C16—H16C	0.9600
C8—H8A	0.9700	S1—O1	1.423 (3)
C8—H8B	0.9700	S1—O2	1.426 (3)
C9—N3	1.268 (3)	S1—N1	1.649 (3)
C9—C10	1.463 (4)	N2—N3	1.405 (3)
C9—H9	0.9300		
			
C2—C1—C6	118.9 (3)	C10—C11—C12	119.8 (3)
C2—C1—N1	119.7 (3)	C10—C11—H11	120.1
C6—C1—N1	121.3 (3)	C12—C11—H11	120.1
C3—C2—C1	121.3 (3)	C13—C12—C11	118.8 (4)
C3—C2—H2	119.4	C13—C12—H12	120.6
C1—C2—H2	119.4	C11—C12—H12	120.6
C2—C3—C4	119.4 (3)	C14—C13—C12	123.5 (3)
C2—C3—H3	120.3	C14—C13—F1	118.2 (4)
C4—C3—H3	120.3	C12—C13—F1	118.4 (4)
C5—C4—C3	120.8 (3)	C13—C14—C15	117.8 (4)
C5—C4—Br1	119.9 (2)	C13—C14—H14	121.1
C3—C4—Br1	119.3 (2)	C15—C14—H14	121.1
C4—C5—C6	120.7 (3)	C14—C15—C10	120.8 (4)
C4—C5—H5	119.7	C14—C15—H15	119.6
C6—C5—H5	119.7	C10—C15—H15	119.6
C5—C6—C1	118.9 (3)	N1—C16—H16A	109.5
C5—C6—C7	118.7 (2)	N1—C16—H16B	109.5
C1—C6—C7	122.4 (2)	H16A—C16—H16B	109.5
N2—C7—C6	118.5 (2)	N1—C16—H16C	109.5
N2—C7—C8	123.1 (2)	H16A—C16—H16C	109.5
C6—C7—C8	118.4 (2)	H16B—C16—H16C	109.5
C7—C8—S1	110.0 (2)	O1—S1—O2	118.54 (17)
C7—C8—H8A	109.7	O1—S1—N1	107.04 (16)
S1—C8—H8A	109.7	O2—S1—N1	110.83 (16)
C7—C8—H8B	109.7	O1—S1—C8	110.49 (17)
S1—C8—H8B	109.7	O2—S1—C8	108.71 (16)
H8A—C8—H8B	108.2	N1—S1—C8	99.57 (14)
N3—C9—C10	121.5 (3)	C1—N1—C16	120.7 (3)
N3—C9—H9	119.2	C1—N1—S1	117.5 (2)
C10—C9—H9	119.2	C16—N1—S1	117.2 (2)
C11—C10—C15	119.4 (3)	C7—N2—N3	113.6 (2)
C11—C10—C9	121.5 (3)	C9—N3—N2	111.9 (2)
C15—C10—C9	119.1 (3)		
			
C6—C1—C2—C3	−1.7 (5)	C11—C12—C13—C14	−0.7 (6)
N1—C1—C2—C3	176.1 (3)	C11—C12—C13—F1	179.5 (3)
C1—C2—C3—C4	1.9 (5)	C12—C13—C14—C15	0.0 (6)
C2—C3—C4—C5	−0.9 (5)	F1—C13—C14—C15	179.8 (3)
C2—C3—C4—Br1	−179.8 (3)	C13—C14—C15—C10	0.9 (6)
C3—C4—C5—C6	−0.3 (5)	C11—C10—C15—C14	−1.2 (5)
Br1—C4—C5—C6	178.6 (2)	C9—C10—C15—C14	178.1 (3)
C4—C5—C6—C1	0.4 (5)	C7—C8—S1—O1	−169.6 (2)
C4—C5—C6—C7	−179.4 (3)	C7—C8—S1—O2	58.7 (3)
C2—C1—C6—C5	0.6 (4)	C7—C8—S1—N1	−57.2 (2)
N1—C1—C6—C5	−177.2 (3)	C2—C1—N1—C16	−3.3 (5)
C2—C1—C6—C7	−179.6 (3)	C6—C1—N1—C16	174.5 (3)
N1—C1—C6—C7	2.6 (5)	C2—C1—N1—S1	152.0 (3)
C5—C6—C7—N2	−9.5 (4)	C6—C1—N1—S1	−30.2 (4)
C1—C6—C7—N2	170.6 (3)	O1—S1—N1—C1	169.3 (3)
C5—C6—C7—C8	170.0 (3)	O2—S1—N1—C1	−60.1 (3)
C1—C6—C7—C8	−9.8 (4)	C8—S1—N1—C1	54.3 (3)
N2—C7—C8—S1	−140.5 (3)	O1—S1—N1—C16	−34.5 (3)
C6—C7—C8—S1	40.0 (3)	O2—S1—N1—C16	96.1 (3)
N3—C9—C10—C11	7.3 (5)	C8—S1—N1—C16	−149.5 (3)
N3—C9—C10—C15	−171.9 (3)	C6—C7—N2—N3	−179.8 (2)
C15—C10—C11—C12	0.5 (5)	C8—C7—N2—N3	0.7 (4)
C9—C10—C11—C12	−178.7 (3)	C10—C9—N3—N2	−179.4 (3)
C10—C11—C12—C13	0.4 (6)	C7—N2—N3—C9	178.9 (3)

### Hydrogen-bond geometry (Å, º)

**Table:**

*D*—H···*A*	*D*—H	H···*A*	*D*···*A*	*D*—H···*A*
C8—H8*B*···O2^i^	0.97	2.56	3.397 (4)	145
C9—H9···O1^ii^	0.93	2.39	3.292 (4)	163

Symmetry codes: (i) −*x*+1, −*y*+1, −*z*; (ii) *x*, *y*+1, *z*.
